# Is Epstein–Barr virus associated with aggressive forms of breast cancer?

**DOI:** 10.1038/bjc.2011.99

**Published:** 2011-03-29

**Authors:** G Khan, P S Philip, M Al Ashari

**Affiliations:** 1 Department of Microbiology and Immunology, Faculty of Medicine and Health Sciences, United Arab Emirates University, Al Alin, United Arab Emirates; 2 Department of Laboratory Medicine, Tawam Hospital, Al Ain, United Arab Emirates

**Sir,**

We read with great interest the paper by Mazouni *et al* (2011) recently published in the *British Journal of Cancer* linking Epstein–Barr virus (EBV) to aggressive forms of breast cancer.

EBV is a lymphotropic herpesvirus aetiologically associated with a number of human malignancies of both epithelial and lymphoid origin. Although numerous studies have been published over the last 10–15 years looking at the possible link between EBV and the pathogenesis of breast cancer, the association remains controversial and reports from both corners of the arena continue to be published (reviewed in Amarante and Watanabe, 2009). It is possible that the discrepancies between the different reports are due to the differing methodologies used for the detection of EBV, the histological types of tumours examined, and the ethnic/geographical background of the cases studied. For example, using PCR-based techniques, a number of studies have reported a positive correlation between EBV and breast cancer, with up to 50% of cases giving a positive signal (Murray *et al*, 2003; Preciado *et al*, 2005; Fawzy *et al*, 2008). However, owing to the fact that EBV is a ubiquitous virus present asymptomatically in over 90% of the world population, its mere detection in tumour tissue cannot be used to imply disease association. In this context, the findings reported by Marzouni *et al* have to be interpreted with caution. Indeed, several studies that have used the EBER-*in situ* hybridisation (EBER-ISH) approach have failed to show an association, even in cases that were EBV PCR positive (Deshpande *et al*, 2002; Herrmann and Niedobitek, 2003; Murray *et al*, 2003; Thorne *et al*, 2005). Similarly, PCR studies on microdissected tumour cells have also led to contradictory findings. While Fina *et al* (2001) reported the presence of EBV in microdissected tumour cells, Murray *et al* (2003) did not find any evidence of the presence of EBV in the microdissected tumour cells of their cases, even though 21% of the cases were EBV positive by quantitative real-time PCR.

We too have recently examined the association of EBV with the pathogenesis of breast cancer in a large series of cases in the Arab population from the United Arab Emirates. A total of 219 samples from 61 cases were examined using an EBER-ISH method capable of detecting as little as a few EBV-infected lymphocytes in an entire tissue section (Khan *et al*, 1992). All but four cases had multiple tissues (both benign and malignant) that were studied. We found that, although EBV can be detected in approximately 50% of breast cancer cases, the virus is not present in the malignant cells. Rather, the virus is localised to occasional infiltrating lymphocytes (Figure 1), which could give rise to ‘false’ interpretations linking the virus to the pathogenesis of the disease. Furthermore, we did not find any correlation between the presence of EBV in infiltrating lymphocytes and ER, PR, HER2 expression or metastasis status. We believe that our findings of EBV in infiltrating lymphocytes could explain some of the controversies relating to the role of EBV in the pathogenesis of breast cancer.

## Figures and Tables

**Figure 1:**
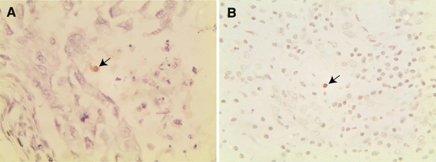
EBER-*in situ* hybridisation for the detection of EBV in breast cancer tissues. (**A**) A metastatic ductal carcinoma from a 39-year-old Emirati woman. This case was triple negative (ER−, PR−, HER2−), but an occasional EBV-positive non-malignant cell could be detected using EBER-*in situ* hybridisation (arrow). (**B**) A case of invasive intraductal beast carcinoma with significant inflammatory reaction from a 43-year-old Emirati woman. EBER-*in situ* hybridisation revealed the presence of EBV in an occasional non-malignant lymphoid cell (arrow).

## References

[bib1] Amarante MK, Watanabe MAE (2009) The possible involvement of virus in breast cancer. J Cancer Res Clin Oncol 135: 329–337 19009309 10.1007/s00432-008-0511-2 PMC12160138

[bib2] Deshpande CG, Badve S, Kidwai N, Longnecker R (2002) Lack of expression of the Epstein-Barr virus (EBV) gene products, EBERs, EBNA1, LMP1, and LMP2A, in breast cancer cells. Lab Invest 82: 1193–1199 12218080 10.1097/01.lab.0000029150.90532.24

[bib3] Fawzy S, Sallam M, Awad NM (2008) Detection of Epstein-Barr virus in breast carcinoma in Egyptian women. Clin Biochem 41: 486–492 18258188 10.1016/j.clinbiochem.2007.12.017

[bib4] Fina F, Romain S, Ouafik L, Palmari J, Ben Ayed F, Benharkat S, Bonnier P, Spyratos F, Foekens JA, Rose C, Buisson M, Gérard H, Reymond MO, Seigneurin JM, Martin PM (2001) Frequency and genome load of Epstein-Barr virus in 509 breast cancers from different geographical areas. Br J Cancer 84: 783–790 11259092 10.1054/bjoc.2000.1672 PMC2363823

[bib5] Herrmann K, Niedobitek G (2003) Lack of evidence for an association of Epstein-Barr virus infection with breast carcinoma. Breast Cancer Res 5: R13–R17 12559053 10.1186/bcr561 PMC154138

[bib6] Khan G, Coates PJ, Gupta RK, Kangro HO, Slavin G (1992) Presence of Epstein-Barr virus in Hodgkin's disease is not exclusive to Reed-Sternberg cells. Am J Pathol 140: 757–762 1314022 PMC1886366

[bib7] Mazouni C, Fina F, Romain S, Ouafik L, Bonnier P, Brandone J, Martin P (2011) Epstein-Barr virus as a marker of biological aggressiveness in breast cancer. Br J Cancer 104: 332–337 21179039 10.1038/sj.bjc.6606048 PMC3031896

[bib8] Murray PG, Lissauer D, Junying J, Davies G, Moore S, Bell A, Timms J, Rowlands D, McConkey C, Reynolds GM, Ghataura S, England D, Caroll R, Young LS (2003) Reactivity with a monoclonal antibody to Epstein-Barr virus (EBV) nuclear antigen 1 defines a subset of aggressive breast cancers in the absence of the EBV genome. Cancer Res 63: 2338–2343 12727860

[bib9] Preciado MV, Chabay PA, De Matteo EN, Gonzalez P, Grinstein S, Actis A, Gass HD (2005) Epstein-Barr virus in breast carcinoma in Argentina. Arch Pathol Lab Med 129: 377–381 15737034 10.5858/2005-129-377-EVIBCI

[bib10] Thorne LB, Ryan JL, Elmore SH, Glaser SL, Gulley ML (2005) Real-time PCR measures Epstein-Barr virus DNA in archival breast adenocarcinomas. Diagn Mol Pathol 14: 29–33 15714061 10.1097/01.pas.0000144448.23464.ab

